# Patterns in network activity and information processing in a detailed computer model of the cerebellar granular layer

**DOI:** 10.1186/1471-2202-15-S1-O2

**Published:** 2014-07-21

**Authors:** Shyam Kumar, Sungho Hong, Erik De Schutter

**Affiliations:** 1 Computational Neuroscience Unit, Okinawa Institute of Science and Technology, Onna-son, Okinawa 904-0895, Japan; 2 Department of Theoretical Neurobiology and Neuroengineering, University of Antwerp, Wilrijk, Belgium 2610

## 

In the cerebellar cortex, the granular layer is at the first stage of processing information from other brain regions delivered via mossy fibers. The major components of this neural network are numerous and tiny granule cells (GrC), which are the only excitatory neurons, and Golgi cells (GoC) that provide the only inhibitory inputs to GrCs. Despite such structural simplicity, many questions about their functions remain unanswered.

**Figure 1:**
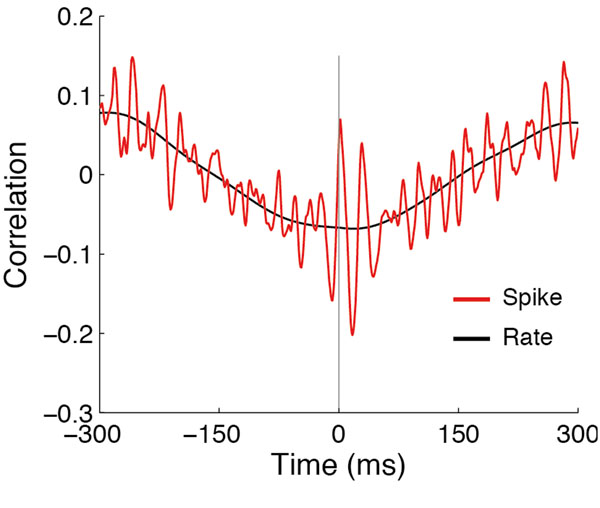
Cross-correlogram between the average GrC activity within the stimulated and unstimulated region (diameter of each region = 200 μm, distance = 500 μm). The stimulated region was given high frequency mossy fiber input of 20 Hz and 50 Hz, each lasting 300 ms. The spike trains were formed with a 1 ms bin, while the rate is evaluated with a 100 ms time window.

Here we investigate the signal transformation property of the granular layer neural network with our three-dimensional large-scale and detailed computer model, composed of the biophysically detailed ~8×10^5^ GrC and ~2000 GoC models with their physiological synaptic and electrical connectivity. With background and constant mossy fiber inputs, the model shows network-wide oscillations driven by the synchronized GoC firing, as in previous simulation and experimental studies [1-3]. Oscillation frequency was usually higher than the Golgi cell-firing rate, as some GoCs exhibited cycle skipping. With more physiological and diverse paradigms of mossy fiber stimulation, we could observe interesting patterns which hint that this oscillation and rate coding synergistically contribute to the network outputs. For example, when the mossy fiber stimulation is spatially limited, there is anti-correlation in the GrC spike count between the stimulated and unstimulated region, suggesting center-surround “receptive fields” [4], while the oscillation persists and opens time windows for well-timed spikes [5] (Figure 1). Our results suggest how the complex dynamics of the granular layer network due to cellular and synaptic properties can contribute to its rich information processing.
